# Detection of Simultaneous Group Effects in MicroRNA Expression and Related Target Gene Sets

**DOI:** 10.1371/journal.pone.0038365

**Published:** 2012-06-19

**Authors:** Stephan Artmann, Klaus Jung, Annalen Bleckmann, Tim Beißbarth

**Affiliations:** 1 Department of Medical Statistics, University Medical Center Göttingen, Göttingen, Germany; 2 Department of Hematology and Oncology, University Medical Center Göttingen, Göttingen, Germany; University of Turin, Italy

## Abstract

Expression levels of mRNAs are among other factors regulated by microRNAs. A particular microRNA can bind specifically to several target mRNAs and lead to their degradation. Expression levels of both, mRNAs and microRNAs, can be obtained by microarray experiments. In order to increase the power of detecting microRNAs that are differentially expressed between two different groups of samples, we incorporate expression levels of their related target gene sets. Group effects are determined individually for each microRNA, and by enrichment tests and global tests for target gene sets. The resulting lists of *p*-values from individual and set-wise testing are combined by means of meta analysis. We propose a new approach to connect microRNA-wise and gene set-wise information by means of *p*-value combination as often used in meta-analysis. In this context, we evaluate the usefulness of different approaches of gene set tests. In a simulation study we reveal that our combination approach is more powerful than microRNA-wise testing alone. Furthermore, we show that combining microRNA-wise results with ‘competitive’ gene set tests maintains a pre-specified false discovery rate. In contrast, a combination with ‘self-contained’ gene set tests can harm the false discovery rate, particularly when gene sets are not disjunct.

## Introduction

### Biological Background and Motivation

Interest in microRNAs (miRNAs) has been continuously growing in recent years [1]. They regulate gene expression and play a role in a wide spectrum of biological fields, ranging from developmental to tumour biology [2]. Their function is to regulate gene expression by down-regulation of mRNAs. The gene transcripts get either directly degraded or their translation is inhibited. This gives reason to search for differentially expressed miRNAs between two different groups of biological samples, which was widely done for mRNAs in the past.

While the exact mode of miRNA action is still under investigation, more and more details of the mechanism of miRNA-mediated inhibition of gene expression are being discovered. It is known, for instance, that their precursors are transcribed in the nucleus. After several processing steps and the export into the cytoplasm a mature miRNA is incorporated into the RNA-induced silencing complex, referred to as ‘RISC’. This protein complex affects the levels of gene expression, while the miRNA itself only acts by guiding the complex to mRNAs based on sequence homology. Consequently, each miRNA has a set of target mRNAs defined by the sequence of both. The target set is supposed to be more or less unique for each miRNA. However, the target sets of two miRNAs need not necessarily be disjunct.

The sequence dependent specificity of miRNAs for their target gene sets can be predicted. Several databases of such predicted target sets have been established, for example ‘microCosm’ [3] and ‘TargetScan’ [4]. Additionally, databases of experimentally validated miRNA-targets exist [5].

Expression levels of both, mRNA-targets and miRNAs, can be detected in a high-throughput manner. Microarrays have been used in the last decade to measure RNA levels. They have been adopted for miRNAs as well and are gaining popularity [6], [7].

The above-mentioned sources of information, i.e. the available links between miRNAs and their related target gene sets through databases as well as observed expression levels through microarray experiments, can provide a close view of a cell’s, tissue’s or organism’s miRNA status. However, searching for differentially expressed miRNAs, has mainly concentrated on miRNA-wise testing so far. In the case that researchers took miRNA target sets into account, this was primarily done separately from miRNA analysis. Mostly, only those target sets were studied whose miRNAs were already detected as differentially expressed. These current approaches can, however, lead to more false positives or negatives than necessary. Assume, for example, a particular miRNA that is highly expressed under a certain condition but whose target mRNAs are absent in a cell anyway. The effect on the cell’s phenotype of such a miRNA can be expected to be rather small. Vice versa, due to the enzymatic function of the RISC complex, little changes in miRNA levels can lead to large effects in their targets’ levels. Thus, many interesting features can be missed when information about target gene sets are omitted.

In order to increase the power of detecting differentially expressed miRNAs in the two-group design, the aim of this work is to combine miRNA- and mRNA-expression data. More precisely, we present and evaluate an approach to detect pairs of miRNAs and related target sets of mRNAs that simultaneously exhibit a group effect. Our approach is to first analyse miRNA and mRNA expression data separately, and then to combine the resulting information. All software used and implemented is written in the R statistical programming language [8].

### Gene-wise and Gene Set Testing

One approach to detect group effects in miRNA expression data is to perform component-wise statistical tests. Each test analyses whether a particular miRNA is differentially expressed between the two groups. A large number of methods exists, for example mixture models [9], permutation tests [10], empirical Bayes approaches [11] and analysis of variance models [12]. Here, we employ a widely used linear model implemented in the R-package ‘limma’ which makes use of empirical Bayes methods [13].

Group effects in the related target gene sets are not studied by individually testing each component of a set. Instead, the group effect is assessed globally for a whole set. Different statistical approaches of gene set tests have been proposed which can be divided into ‘self-contained’ and ‘competitive’ tests [14]. The self-contained approaches incorporate only expression levels of the genes of a particular set. As self-contained methods we will employ and compare so called global tests as well as a rotation-based procedure. Competitive approaches, on the other hand, incorporate the expression levels of all genes within a microarray experiment. ‘Gene Set Enrichment Analysis’ (GSEA) [15], for example, studies the distribution of ranks of differentially expressed genes from the target gene set using a weighted form of the Kolmogorov-Smirnov test.

**Figure 1:**
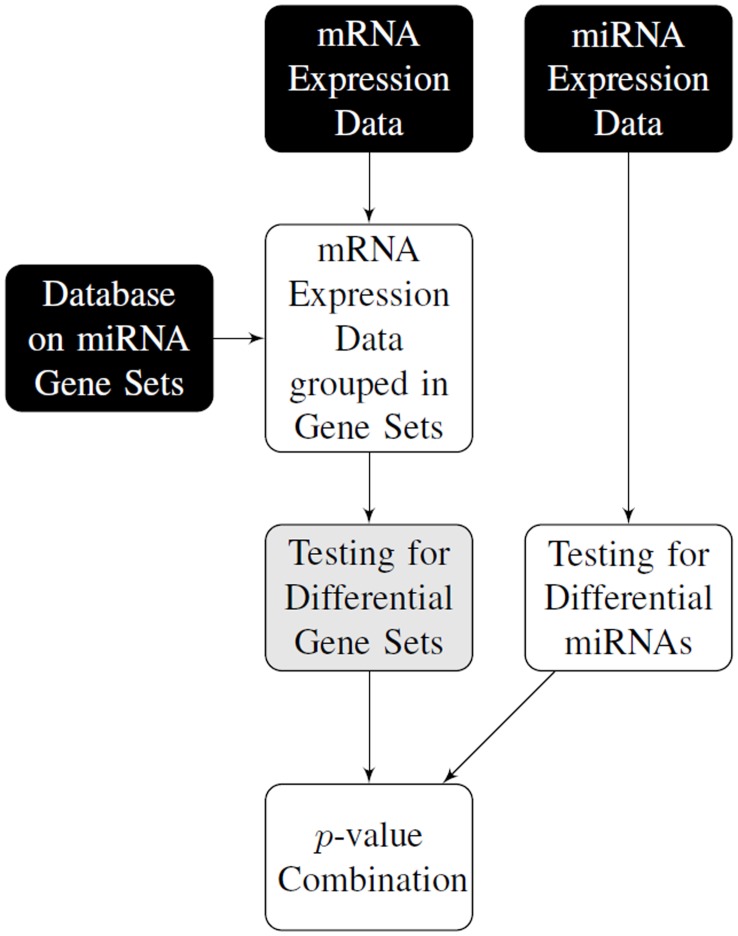
Flow chart of combining expression levels of miRNAs and their related target mRNAs. The links between microRNAs and their target mRNAs are taken from public databases. MicroRNAs and target sets are first analysed separately and obtained *p*-values are combined as final result.

Component-wise testing of miRNAs and simultaneous testing of related target gene sets yields two lists of *p*-values. In order to detect which (miRNA, gene set)-pairs possess a simultaneous group effect we propose a new approach. In particular, we employ methodology frequently used in the field of meta analysis to combine *p*-values from different experiments. Such methods were already used for gene expression data, however, in a different context than ours, for example to combine microarray results from different experimental stages [16]. A flow chart of our approach is depicted in Figure 1. The combined *p*-values are finally translated into a score.

In the following paragraph we detail the methods for component-wise testing, gene set testing and *p*-value combination. Subsequently we show the results of a simulation study. In this simulation study we determined the *false-discovery rate* (*FDR*), i.e. the portion of false positive detections among all positive test results, with regard to simultaneous group effect of miRNAs and related target gene sets. Additionally, we determined the *average power rate* (*APR*), i.e. the portion of all true positive detections. Besides, we evaluate the performance of our combination approach on one example of mouse expression data and one of expression in HIV samples. Finally, the results are discussed.

## Methods

### MicroRNA-wise Testing

For miRNA-wise testing we apply the linear models proposed by [13]. They can generally be used for analysing gene expression data in dependence from several experimental factors. In the particular design we are studying here, i.e. one group factor with two levels, the group effect of gene 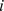 (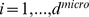) can be denoted by 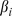 in these models. Consequently, the related model tests separately for each gene the null hypothesis of no group effect, i.e. 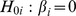.

According to [13], because of the large number of hypotheses, these are tested by moderated *t*-type statistics. These test statistics are based on the fact that for some features a very small variance is expected although the related difference of group means is rather small. Thus, the ordinary *t*-statistic would become unreasonable large for these features. The moderated *t*-statistic is calculated by incorporating a prior distribution for the standard deviations of the components and shrinking the observed standard deviation to these prior values. In our simulations and data analyses, we use the R-package ‘limma’ to test each individual hypothesis by the moderated *t*-statistic, obtaining one *p*-value per miRNA.

### Gene Set Testing

#### Self-contained methods

In order to test the group effects within the target mRNA sets, we use different so called global tests. Each of them forms a self-contained method, since only the expression levels of the target set but no other genes of the microarray experiment are used. The first one is the ‘globaltest’ procedure proposed by [17]. This procedure is based on a logistic regression model. Thus, the group factor exhibits a dichotomous response parameter 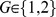 and one tests the null hypothesis that the group membership is independent from the gene expression, i.e. 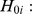 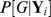  =  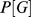. Here, 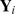 denotes the matrix of expression levels for the 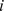
th target gene set. The test statistic for this model is asymptotically normally distributed. For large sample sizes a sample permutation approach is offered as well. Another approach (‘GlobalAncova’) was proposed by [18] and improved by [19]. This approach uses an ANCOVA-model testing the reverse null hypothesis, i.e. that the gene expression is independent from the group membership. This hypothesis can be formulated by 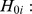 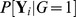  =  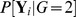. This approach also offers an asymptotic result and a permutation algorithm as well. The third approach of global testing is a general model for high-dimensional repeated measures data [20], [21], in the following referred to as ‘RepeatedHighDim’. This approach tests the same hypothesis as ‘GlobalAncova’. All three approaches, ‘globaltest’, ‘GlobalAncova’ and ‘RepeatedHighDim’ are implemented in R-packages of the same names.

As another self-contained method, we regard the ‘ROAST’ test proposed by [22] also implemented in the R-package ‘limma’. In this approach, the data is modelled similarly to the above-detailed models of [13]. The moderated *t*-statistics are calculated mRNA-wise and transformed to standard normal variables. A gene set statistic is then calculated by summarizing the mRNA-wise statistics. Here we use the unweighted mean of the *t*-statistics. To account for inter-gene correlations, random rotations [23] are applied and an exact *p*-value is calculated.

#### Competitive methods

The competitive gene set methods we investigate are similar to GSEA proposed by [15]. Using data from a whole microarray experiment we employ again the models of [13] ‘limma’-package to produce mRNA-wise *p*-values with regard to differential expression. A rank is assigned to each mRNA according to the position of its *p*-value. Three different enrichment tests are applied to analyse the distribution of ranks belonging to a particular gene set. The first one is the one-sided Kolmogorov-Smirnov test. It is used to test the null hypothesis that the ranks of the genes inside the gene set come from a uniform distribution. The alternative is that the distribution of the genes in the gene set is skewed towards lower ranks. As second approach, the two-sample Wilcoxon test is applied on gene ranks. It compares the distribution of ranks inside and outside the gene set. Finally, a method using a 2×2-contingency table is employed. In particular, Fisher’s exact test is employed to compare the proportion of differentially expressed genes among the gene set with the proportion of differentially expressed genes outside the gene set.

In addition, we employ a rotation test, that is the competitive ‘Romer’ test [24]. It is similar to the above-mentioned ‘ROAST’ test, but it is competitive in that it compares gene sets. For both tests we set the number of rotations to 1000 to be able to produce *p*-values as low as 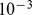.

### Combination of *p*-values

While component-wise testing yields one *p*-value per miRNA, gene set testing yields one *p*-value for each of the related gene sets. We use methodology often used in meta analysis to bring this information together. The behaviour of a miRNA and its related gene set is usually supposed to be inverse, i.e. up-regulation of a particular miRNA is supposed to cause a down-regulation of the related gene set. Accordingly, we are particularly interested in detecting these cases in our data analyses. Therefore, we perform gene set testing in a one-sided way, one time in each direction. We denote the *p*-values for the alternative hypotheses of up- and down-regulation of an miRNA by 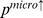 and 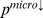, respectively. The related *p*-values for up- and down-regulation of a target gene set are denoted by 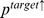 and 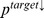, respectively.

One-sided hypotheses can be tested for the competitive enrichment tests, i.e. for the Kolmogorov-Smirnov, the Wilcoxon and Fisher’s exact test. One-sided testing is also possible for the two rotation test, i.e. for the competitive Romer and the self-contained ROAST test. The hypotheses for the three self-contained global tests, i.e. globaltest, GlobalAncova and RepeatedHighDim, however, can not be reasonably stated in a one-sided way. Therefore, we split the target gene sets when using these global procedures and test the two sets of up- and down-regulated genes separately. The direction of regulation is determined for each gene by comparison of group means. It should be mentioned that this approach for the self-contained tests may introduce some bias as the observed data itself is used for the prior partitioning. However, the results of our simulations below show that our methods yet maintain a prespecified 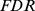 in most situations.

For the combination of *p*-values we distinguish two cases. For the two rotation tests as well as for the Wilcoxon test the target *p*-values for up- and down-regulation (approximately for the rotation tests) sum up to one, i.e. 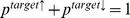. In this case we use Stouffer’s inverse normal method [16], [25] to combine *p*-values. For all other gene set tests in most cases we obtain 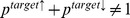, and we will use Fisher’s combination method then [26]. In both cases, we first combine 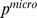 and 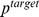, separately for up- and down-regulation, and derive then a final score based on the two combined *p*-values. The final results should rather be regarded as scores than as a *p*-values, although these scores range from zero to one. The calculation of the scores is described in the following.

#### Fisher’s combination method

Because our primary goal is to detect differentially expressed miRNAs using the additional information about their target’s regulation, and because we assume miRNA and target regulation to be inverse, we combine the miRNA’s *p*-value of up-regulation with the target’s *p*-value for down-regulation and vice versa. According to Fisher’s method, the combined *p*-values for the *i*th miRNA are given by
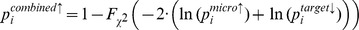
and



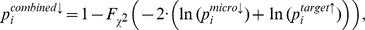
where 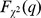 is the 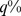-quantile of the 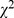-distribution with four degrees of freedom.

In order to obtain a decision value between zreo and one, we build as final score
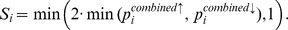



#### Stouffer’s inverse normal method

According to Stouffer’s inverse normal method the combination is performed as follows:
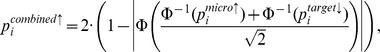
and



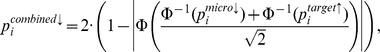
where 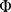 is the cumulative distribution function of the standard normal distribution. Here, we derive the final score by



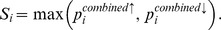



Note that when Stouffer’s method is applied for the Wiloxon-test as the gene set testing procedure, the scores 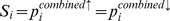 can be regarded as *p*-values [16]. For the rotation tests, we obtain 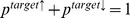 only approximately for large numbers of rotations. As a consequence, [16] 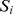 for rotation tests is always more conservative than a *p*-value would be.

### Simulation Study

In order to evaluate whether the above-detailed methods are applicable to detect simultaneous group effects in miRNA and target mRNA expression data, simulation studies that picture the two group design were performed. The numbers of biological replicates per group were set to be 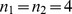. Thus, a total number of 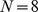 samples was produced per simulation run. The numbers of simulated miRNAs and mRNAs was 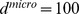 and 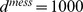, respectively. Expression data were stored in matrices with columns representing samples and rows representing either miRNAs or mRNAs. Data generation was performed as follows.

Expression levels were drawn from multivariate normal distributions 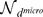 and 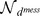, respectively. Mean vectors were drawn from a log-normal distribution and covariance matrices were chosen to have either a block structure, an autoregressive structure or to be of an unstructured type. To generate differentially expressed features 10% of miRNAs and 5% of mRNAs were randomly selected. Half of these features was up-regulated by adding a log fold change of 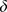 to the corresponding mean vector while the other half was down-regulated by subtracting 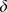.

In order to model the effect of miRNAs on mRNA degradation a 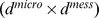 allocation matrix 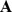 was constructed that defined the targets of each miRNA. Each entry 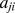 of 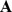 took on the value 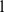 if miRNA 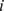 was designated to attack mRNA 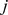 (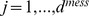), and the value 0 otherwise. Different structures of 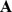 were compared in order to simulate non-overlapping and non-oberlapping gene sets, respectively.

The miRNA effect on mRNA expression levels was constructed by a simple linear model. Denote 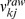 to be the *j*th entry of the mean vector for the mRNAs in group 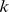 (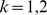), as constructed above. Furthermore, denote 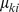 to be the 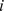
th entry of the mean vector for the miRNAs of the same group. The modifications of the mean vectors 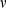 of the mRNA were then modelled in dependence of the mean vectors 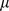 of the miRNAs using the following equation:
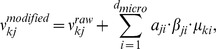
where 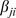 reflects the strength of the modification. The indicator variables 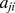 are taken from the allocation matrix 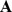. The modification factors 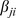 were drawn from univariate normal distributions. The modified expectation vectors 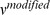 were next used to draw the expression levels of the mRNAs.

In each of 1000 simulation runs expression data were drawn as described above, and the different methods for miRNA-wise testing and gene set testing were carried out. Scores were constructed from the resulting combined 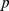-values. After adjusting the scores for multiple hypothesis testing according to [27] the 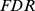 and the 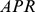 were determined in each simulation run. For all simulated 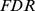
s we evaluated whether they maintain a predefined level of 5%.

### Pathway Analysis of Data Examples

In the analysis of the data examles below, we searched for enriched gene ontology-terms (GO-terms) in order to evaluate which functional role the newly predicted miRNAs play in studied biological context. GO-terms were aquired via the R-packages ‘biomaRt’. For the miRNAs of interest, GO-analysis counts the occurence of terms among the related target genes and compares this to the count in the genes of the not in the target set. The counts are compared between target genes and non-target genes by means of Fisher’s exact test.

## Results

### Simulated False-discovery Rates

In each simulation setting, the 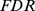 was separately determined 1) for using only the miRNA-wise testing procedure, 2) for using only the testing procedures of the related target gene sets and 3) for using the approach of combined *p*-values. In each of the three variants, the 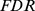 was regarded as the portion of false positively detected miRNAs among all positively detected miRNAs. An overview of the obtained 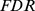 under all simulation conditions for the two latter approaches is given in Tables 1 and 2. No systematic effect could be observed with regard to the different covariance matrices. While the miRNA-wise testing maintained the pre-specified 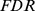-level of 5% within an acceptable range, target set testing and combined testing sometimes exceeded this level. This happened only sometimes for competitive tests, but quite often when self-contained ones were applied.

**Table 1:** Simulated 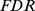 for microRNA-selection based on target set testing.

Gene set test	Covariance matrix	Non-overlap. target sets	Overlap. target sets
GlobalTest	Autoregressive	**0.102**–**0.105**–**0.139**	**0.305**–**0.900**–**0.900**
	Block	**0.052**–**0.107**–**0.124**	0.035–**0.900**–**0.900**
	Unstructured	**0.212**–**0.215**–**0.617**	**0.750**–**0.900**–**0.900**
GlobalAncova	Autoregressive	0.000–0.000–**0.051**	0.04–0.046–**0.058**
	Block	0.002–0.005–**0.053**	0.028–0.031–0.032
	Unstructured	0.000–0.000–0.003	0.000–0.036–0.039
RepeatedHighDim	Autoregressive	0.019–**0.081**–**0.083**	**0.103**–**0.900**–**0.900**
	Block	0.000–0.002–0.011	0.008–0.040–0.049
	Unstructured	0.030–0.034–0.037	0.031–**0.878**–**0.900**
ROAST	Autoregressive	**0.068**–**0.074**–**0.077**	**0.091**–**0.892**–**0.900**
	Block	**0.082**–**0.088**–**0.100**	**0.065**–**0.846**–**0.900**
	Unstructured	0.000–0.002–0.041	0.043–0.046–0.049
KS	Autoregressive	0.002–0.005–0.036	0.029–0.031–0.036
	Block	**0.000**–**0.000**–**0.003**	**0.003**–**0.036**–**0.040**
	Unstructured	0.029–0.031–0.033	0.030–**0.730**–**0.900**
Wilcoxon	Autoregressive	0.001–0.002–0.009	0.010–0.030–0.039
	Block	0.026–0.029–0.035	0.038–**0.882**–**0.900**
	Unstructured	**0.074**–**0.079**–**0.111**	**0.114**–**0.893**–**0.900**
Fisher	Autoregressive	**0.105**–**0.110**–**0.178**	**0.102**–**0.862**–**0.900**
	Block	0.000–0.001–**0.056**	0.042–0.047–0.050
	Unstructured	0.002–0.004–0.049	0.029–0.030–0.033
Romer	Autoregressive	0.000–0.000–0.003	0.003–0.038–0.040
	Block	0.023–0.025–0.031	0.033–**0.741**–**0.900**
	Unstructured	0.000–0.002–0.007	0.005–0.027–0.033

Simulated 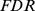 with respect to the type of gene set test and covariance matrix using the approach of selecting microRNAs by testing their target gene sets. Results are presented for the simulation setting of overlapping and disjunct target sets. Presented numbers are the minimum, median and maximum simulated 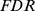 across the range of the log fold change 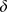 (between 0 and 6). Rates larger than the pre-specified level of 0.05 are printed in bold.

**Table 2:** Simulated 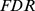 for microRNA-selection based on combined target set and microRNA-wise testing.

Gene set test	Covariance matrix	Non-overlap. target sets	Overlap. target sets
GlobalTest	Autoregressive	**0.069**–**0.074**–**0.095**	**0.149**–**0.900**–**0.900**
	Block	**0.064**–**0.069**–**0.071**	**0.120**–**0.900**–**0.900**
	Unstructured	**0.178**–**0.183**–**0.524**	**0.693**–**0.900**–**0.900**
GlobalAncova	Autoregressive	0.029–0.033–**0.062**	0.044–0.046–**0.062**
	Block	0.043–0.045–**0.063**	0.034–0.036–0.045
	Unstructured	0.005–0.006–0.006	0.007–0.037–0.042
RepeatedHighDim	Autoregressive	0.044–0.047–**0.053**	**0.058**–**0.318**–**0.576**
	Block	0.031–0.034–0.047	0.029–0.037–0.044
	Unstructured	0.033–0.038–0.044	0.027–**0.680**–**0.900**
ROAST	Autoregressive	0.045–0.048–**0.057**	0.035–**0.754**–**0.900**
	Block	**0.095**–**0.105**–**0.127**	**0.075**–**0.814**–**0.900**
	Unstructured	0.033–0.036–**0.057**	0.049–**0.051**–**0.055**
KS	Autoregressive	**0.052**–**0.054**–**0.061**	0.036–0.044–0.045
	Block	**0.010**–**0.012**–**0.013**	**0.013**–**0.047**–**0.049**
	Unstructured	0.037–0.040–0.044	0.025–0.036–0.046
Wilcoxon	Autoregressive	0.039–0.044–**0.058**	0.037–0.039–0.046
	Block	0.026–0.029–0.036	0.030–**0.704**–**0.900**
	Unstructured	0.042–0.050–**0.060**	**0.054**–**0.791**–**0.900**
Fisher	Autoregressive	**0.111**–**0.119**–**0.211**	**0.120**–**0.838**–**0.900**
	Block	0.031–0.034–**0.052**	0.044–0.049–**0.080**
	Unstructured	0.045–0.050–**0.059**	0.039–0.043–**0.054**
Romer	Autoregressive	0.007–0.008–0.012	0.012–0.044–0.047
	Block	0.032–0.034–0.043	0.028–0.039–**0.052**
	Unstructured	0.038–0.041–0.046	0.033–0.036–**0.063**

Simulated 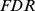 with respect to the type of gene set test and covariance matrix using the approach of combined target set and microRNA-wise testing. Results are presented for the simulation setting of overlapping and disjunct target sets. Presented numbers are the minimum, median and maximum simulated 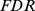 across the range of the log fold change 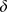 (between 0 and 6). Rates larger than the pre-specified level of 0.05 are printed in bold.

#### Non-overlapping gene sets

In the case of non-overlapping target gene sets, the approach of miRNA-selection based on target set testing became too liberal in many cases when the self-contained tests were applied. In comparison the 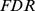 was rather conservative when competitive tests were employed.

A similar result can be observed when selecting miRNAs by combined testing. With this approach the ‘ROAST’ and ‘Romer’ method behave very well, yielding simulated 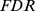
s of 0.032–0.053 and 0.031–0.047, respectively.

#### Overlapping gene sets

**Figure 2:**
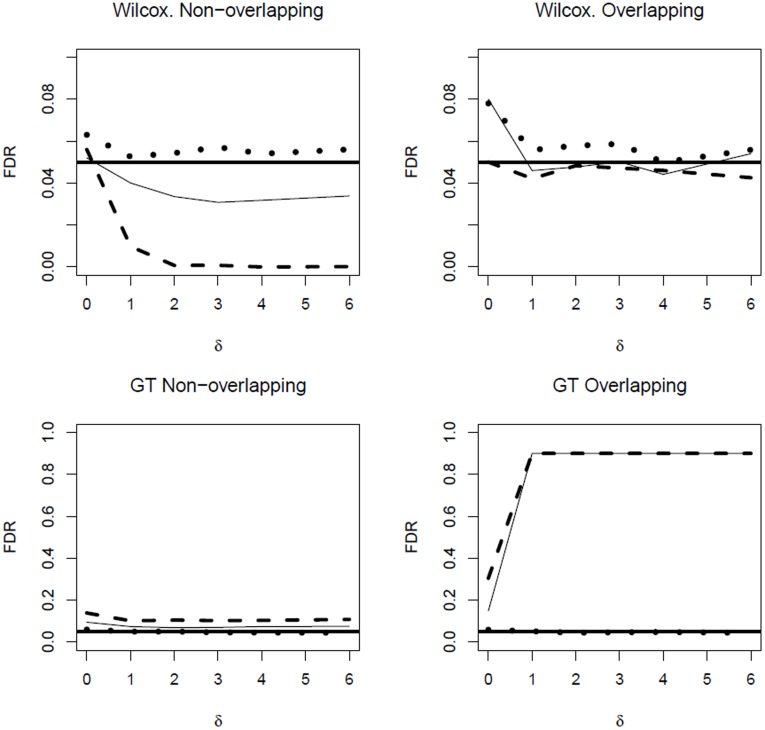
Effect of overlapping target gene sets on the simulated 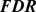. Effects are presented for component-wise testing (dotted line), target set-wise testing (dashed line) and the combination approach (solid line). While the competitive approaches such as the ‘Wilcoxon’-based gene set test (top) still maintained the pre-specified 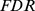-level of 5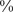 when target gene sets overlapped, the 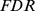 increased dramatically when employing the self-contained approaches such as the ‘globaltest’ procedure (bottom).

Allowing target gene sets to be overlapped increased the simulated 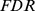 in most of the simulations. Exemplarily, this effect is illustrated in Figure 2 for the Wilcoxon based target set testing (top) and the ‘globaltest’ approach (bottom), where the obtained 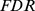 is plotted versus an increasing log fold change. Particularly ‘globaltest’ was very strong affected by letting target sets being overlapped. The 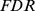 increased very fast with increasing fold changes, up to levels of about 90 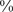, then.

This strong effect could also be observed for the other self-contained tests except for ‘ROAST’. In comparison, the competitive tests still maintained the pre-specified 5%-level in most cases of overlapping target sets. Here, the largest 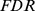 observed was 0.08 (combined testing with the Wilcocon approach).

### Simulated Average Power Rates

In our simulation study the average power rate of each approach was the portion of true positive detected miRNAs among all positives detected miRNAs. Table 3 compares the power rate curves of 1) component-wise miRNA testing, 2) target gene set testing and the 3) combination approach in the different simulation settings. Although, the power curves sometimes intersected, this table gives the general tendency of the relations between the three approaches.

**Table 3:** Relation between simulated power rate curves.

Gene set test	Covariance matrix	Non-overlap. target sets	Overlap. target sets
Globaltest	Autoregr.	miRNA < set ≈ combined	miRNA < set ≈ combined
	Block & unstr.	miRNA < set < combined	miRNA < combined < set
GlobalAncova	Autoregr.	miRNA < set < combined	miRNA < set ≈ combined
	Block & unstr.	miRNA < set < combined	miRNA < combined < set
Rep’HighDim	Autoregr.	miRNA < set ≈ combined	miRNA < set ≈ combined
	Block & unstr.	miRNA < set ≈ combined	miRNA < set < combined
ROAST	Autoregr.	*miRNA < combined < set	miRNA < combined < set
	Block	miRNA ≈ set < combined	miRNA < combined < set
	Unstr.	miRNA ≈ set < combined	miRNA < set ≈ combined
KS	Autoregr.	miRNA < set < combined	miRNA < set < combined
	Block & unstr.	miRNA < set ≤ combined	miRNA < set < combined
Wilcoxon	Autoregr.	miRNA < set < combined	miRNA < set < combined
	Block & unstr.	miRNA < set ≤ combined	miRNA < set < combined
Fisher	Autoregr.	miRNA < set < combined	miRNA < set < combined
	Block & unstr.	miRNA < set ≤ combined	miRNA < set < combined
Romer	Autoregr.	miRNA < combined < set	miRNA < combined < set
	Block & unstr.	set < miRNA < combined	set < miRNA < combined

**Figure 3:**
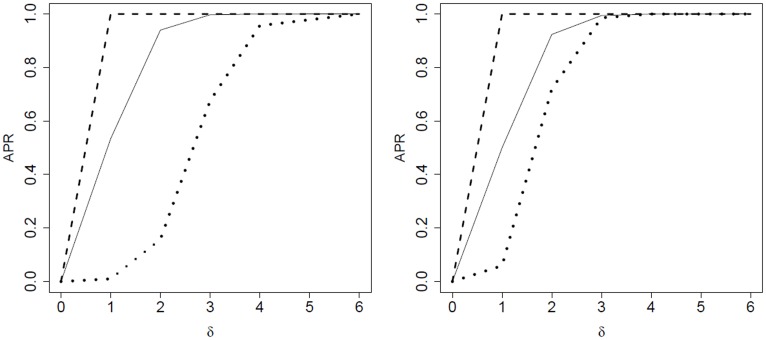
Simulated average power rates with respect to the log fold change 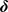. Left: component-wise testing (dotted line), target testing with ‘ROAST’ (dashed line) and the combination approach (solid line), each in the case of an autoregressive covariance structure and non-overlapping target sets. Right: combination approach based on ‘globaltest’ (dotted line), ‘Wilcoxon’ (dashed line) and ‘Romer’ (solid line), each in the case of an unstructured covariance matrix and overlapping target sets.

Relation between simulated power rate curves of microRNA-wise, target set-wise and combined testing. Although, power curves sometimes intersected, this table gives the general tendency of the relations between the three approaches. *Compare Figure 3 left.

#### Non-overlapping gene sets

In most cases of non-overlapping gene sets, component-wise testing of miRNAs resulted in the lowest 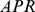, compared to simultaneous testing of the related target gene sets or to the combination approach. Among the latter two approaches, the combination approach mostly yielded the largest power. In some cases, however, pure gene set testing yielded the largest power: as a typical example, the left-hand side of Figure 3 compares the 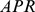 for component-wise miRNA testing, for gene set testing using the ‘ROAST’ method and for the 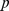-value combination method.

#### Overlapping gene sets

In the case of overlapping target gene sets the performance of competitive combined testing was not seriously affected. For some self-contained tests, however, the combination-approach became worse than testing target sets alone. MiRNA-wise testing, however, was outperformed by the two other approaches in each case.

#### General comparison of 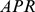


Most often the algorithms based on competitive gene set tests had a higher 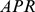 than those based on self-contained tests. The same is true when the performance of the gene set tests on their own is compared. Comparing directly the gene set tests among each other, they sometimes varied greatly (see Table 4). In general, combined testing based on Wilcoxon and Kolmogorow-Smirnov tests had a better performance than the rest. Take Figure 3 on the right for example. There, the 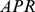 of combined miRNA-wise testing and the Wilcoxon test is clearly higher than for the Romer- and the ROAST-tests.

**Table 4:** Rating orders of the 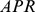 in simulations of combined testing.

Gene sets	Covariance matrix	GT	GA	RHD	KS	W	F	Ro	R
Non-Overlap.	Autoregressive	4	5	3	1	1	8	6	7
	Block	7	8	4	1	1	5	6	3
	Unstructured	7	8	4	1	1	5	6	3
Overlapping	Autoregressive	1	1	1	1	1	6	7	8
	Block	6	6	4	1	1	3	8	5
	Unstructured	7	6	4	1	1	3	8	4

Rating orders are based on the following gene set tests: GlobalTest (GT), GlobalAncova (GA), RepeatedHighDim (RHD), Kolm. Smirnov (KS), Wilcoxon (W), Fisher (F), ROAST (Ro) and Romer (R). The rank 1 denotes the largest power rate while the rank 8 denotes the worst power rate (comparable 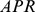-curves were given the same position in the ranking). Note that W and KS always have the largest power rates.

### Analysis of Neurogenesis Data Example

In a data example of miRNA and mRNA expression in rat brains [28] we want to illustrate the gain of using the above detailed combination methods. These example data were obtained from MIAME compliant databases. In particular, miRNA-data were retrieved from ‘ArrayExpress’ [29] (accession number: E-MEXP-1596), the corresponding preprocessed mRNA-data were downloaded from ‘Gene Expression Omnibus’ [30] (GEO accession number: GSE11334). Expression data was subsequently log-transformed and normalised using the quantile method [31]. Data from ‘TargetScan 4.1′ [4] was used to create the allocation matrix 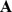.

The example data contains miRNA and mRNA expression profiles from neuronal progenitors isolated from rat brains at embryonic day 11 (E11) and embryonic day 13 (E13). Three animals per group were sacrificed for miRNA arrays while 4 biological replicates were taken for mRNA profiling. Here we will compare expression profiles of early neuronal progenitors (ENPs) in E11 and E13 samples.

Table 5 lists the miRNAs originally detected by Nielsen *et al.* [28] and the results of our reanalysis. The listed miRNAs were reported to be either significantly up- or down-regulated (column 1) with a log fold change larger than 2. For the differential miRNAs, the authors also determined target gene sets with more genes differentially expressed than expected by chance using Fisher’s exact test. This was separately done for up- and down-regulated subsets of each target set (columns 3 and 4). For 20 of the listed miRNAs Nielsen *et al.* find lower 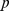-values for the subset of inversely regulated target genes than for those regulated in the same direction as the miRNA, just as one would expect for gene sets attacked by a miRNA. Twelve of the listed miRNAs have a significantly inversely regulated target set according to Nielsen *et al.*

**Table 5:** Original results and re-analysis of microRNAs in rat brains.

	Original analysis			Re-analysis		
miRNA	miRNA	Targ.up	Targ. down	Glob. tests	Enrich. tests	Rot. tests
*miR-99a/b*	up	n.s.	sig.	sig.	sig.	sig.
*miR-9*	up	n.s.	sig.	sig.	sig.	sig.
*miR-100*	up	n.s.	sig.	sig.	sig.	sig.
*miR-181b*/c	up	n.s.	sig.	sig.	sig.	sig.
*miR-125a/b*	up	n.s	n.s	sig.	sig.	sig.
*miR-222*	down	sig.	n.s.	sig.	sig.	sig.
*miR-291-3p*	down	sig.	n.s.	sig.	sig.	sig.
*miR-92*	down	sig.	n.s.	sig.	sig.	sig.
*miR-145*	down	sig.	n.s.	sig.	sig.	sig.
*miR-183*	down	sig.	n.s.	sig.	sig.	sig.
*miR-363-3p*	down	sig.	sig.	sig.	sig.	sig.
*miR-143*	down	n.s.	n.s.	sig.	sig.	sig.
*miR-200b/c*	down	n.s.	n.s.	sig.	sig.	sig.
*miR-20b*	down	n.s.	n.s.	sig.	sig.	sig.
*miR-219*	down	n.s.	n.s.	sig.	sig.	sig.
*miR-18*	down	n.s.	n.s.	sig.	sig.	sig.
*miR-205*	down	n.s.	n.s.	sig.	sig.	sig.
*miR-292-3p*	down	n.s.	n.s.	sig.	sig.	sig.
*miR-218*	up	sig.	n.s.	sig.	sig.	n.s.
*miR-7*	up	n.s.	n.s.	sig.	sig.	n.s.
*miR-124a*	up	n.s.	sig.	sig.	sig.	n.s.
*miR-214*	down	n.s.	n.s.	sig.	sig.	n.s.
*miR-199a*	down			sig.	sig.	n.s.
*miR-19a*	down	sig.	n.s.	sig.	sig.	sig. (R)
*miR-210*	down	n.s.	n.s.	sig.	sig.	sig. (R)
*miR-126*	down	n.s.	n.s.	sig.	sig.	sig. (r)
*miR-290*	down	n.s.	sig.	sig.	sig.	n.s.

MicroRNAs originally detected in expression data from rat brains by Nielsen *et al.* [28]. Columns from left to right are the name of the microRNAs, the originally reported results (columns 2-4) and the results of our re-analysis (columns 5-7). Nielsen *et al.* performed their gene set tests separately for subsets of up- and down-regulated mRNAs yielding two results per gene set. Our re-analysis was performed by use of the combination approach based on either global tests, enrichment tests or rotation tests (‘ROAST’ (R), ‘Romer’ (r)). Significance (sig.) was declared when the FDR-adjusted 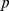-value was 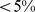 and not significant (n. s.) otherwise.

For our re-analysis data of 223 miRNAs were available, where 202 of them were associated with a target gene set of at least two mRNAs (according to the ‘TargetScan’ 4.1 database). We applied our different approaches to the expression levels of these 202 (miRNA, target set)-pairs.

Results of our re-analysis are also included in Table 5. The uppermost listed 18 miRNAs were determined significant by our combination approach based on either the global tests, the enrichment tests or the rotation tests (columns 5 to 7).

The following listed five miRNAs were not detected by our rotation test-based combination approach, but by the approaches based on global tests and enrichment tests. One of these five, miR-218, even had a gene set differential in the same direction as the miRNA in the original publication.

Among the four bottom-listed features, *miR-19a*, *miR-210* and *miR-126* were significant by our global test- and enrichment test-based approach but only by one of the two rotation-based methods (either ‘Romer’ or ‘ROAST’). We add, that these miRNA also had only a weak fold change.

Out of the miRNAs originally detected by Nielsen *et al.*, miR-290 got the lowest number of procedures to declare it significant in our re-analysis. Indeed, in their original analysis the authors point out that its target gene set tended to be regulated in the same direction as the miRNA itself.

As far as the remaining miRNAs are concerned, eighteen were significant in all tests applied. All of these were also differentially expressed according to miRNA-wise testing. ‘Romer’, as a rather conservative test, detected just three more miRNAs, ‘ROAST’ already 25, the ‘KS’, W’- and ‘Fisher’-based Tests 36, 45 and 76, respectively, and the Global Test-based procedures showed a rather liberal behaviour. The ‘globaltest’, ‘GlobalAncova’ and ‘RepeatedHighDim’-based methods detected all but 31, 34 and 35 miRNAs, respectively.

Our GO-analysis (see Table 6) for the eighteen additional miRNAs detected by all approaches returned 285 (out of 12225) GO-terms that were significantly correlated with the related target sets. Among the top scoring GO-categories mainly terms regarding transcription were detected, however other terms concerning the topic under investigation appear as well. Especially, we find terms related to transcription factor activity (e.g. ‘sequence-specific DNA binding transcription factor activity’, ‘regulation of transcription, DNA-dependent’, ‘positive regulation of transcription from RNA polymerase II promoter’ among the top three terms), related to development (e.g. ‘growth factor binding’, ‘anterior/posterior pattern specification’ or ‘in utero embryonic development’ on positions 11, 15, 16), and related to neurogenesis (e.g. ‘axon guidance’ and ‘axonogenesis’ on positions 8 and 24.) All of these pathways play a role in the development of neurons.

**Table 6:** GO-Terms for Data Examples.

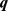 -value	GO-Term
**Rat Neurogenesis**	
7.42e-20	sequence-specific DNA binding transcription factor activity
1.71e-17	regulation of transcription, DNA-dependent
6.90e-17	positive regulation of transcription from RNA polymerase II promoter
8.57e-13	sequence-specific DNA binding
2.92e-12	transcription, DNA-dependent
2.32e-10	negative regulation of transcription from RNA polymerase II promoter
3.00e-10	zinc ion binding
1.64e-09	axon guidance
1.64e-09	DNA binding
6.79e-09	SMAD binding
1.02e-08	growth factor binding
7.56e-08	negative regulation of transcription, DNA-dependent
2.92e-07	RNA polymerase II core promoter proximal
	region sequence-specific DNA binding transcription factor
	activity involved in positive regulation of transcription
2.63e-06	positive regulation of transcription, DNA-dependent
4.72e-06	anterior/posterior pattern specification
5.36e-06	in utero embryonic development
7.85e-06	regulation of actin cytoskeleton organization
1.05e-05	ephrin receptor binding
1.37e-05	gastrulation with mouth forming second
1.42e-05	regulation of translation
1.43e-05	regulation of cell-matrix adhesion
1.85e-05	palate development
2.51e-05	transmembrane receptor protein serine/threonine kinase activity
2.99e-05	axonogenesis
**HIV-dataset**	
3.23e-15	S phase
4.21e-14	regulation of gene silencing
1.36e-13	nucleosome
8.53e-12	nucleoplasm
5.86e-11	nucleosome assembly
4.70e-10	negative regulation of megakaryocyte differentiation
3.24e-09	chromosome
1.53e-05	transcription initiation, DNA-dependent
0.0002	telomere maintenance
0.0002	chromatin organization
0.0009	phosphatidylinositol-mediated signaling
0.0025	CenH3-containing nucleosome assembly at centromere
0.0119	gene expression
0.0279	ribonucleoprotein complex
0.0296	blood coagulation
0.0420	protein refolding
0.0487	signalosome

The top-scoring Gene Ontology (GO) terms with lowest p-values (according to one-sided Fisher’s exact test) of miRNAs’ target sets from Neurogenesis (above) and HIV (below) data example.

### Analysis of HIV Data Example

For further evaluation of our proposed methods we apply them on another example of parallel mRNA and miRNA data. In particular, we analyse data published by Gupta et al. [32] who compared normal primary peripheral blood mononuclear cells to such infected with HIV. Their miRNA and mRNA are available from GEO (accession numbers: GSE33877 and GSE33837).

In this dataset, the rather conservative yet reliable rotation-test-based approach fails to find any miRNAs, i.e. there were no 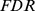-adjusted scores 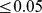, while self-contained tests returned large numbers of significant miRNAs. Therefore, we rely in the following on the Wilcoxon test-based approach, which has presented itself to be less conservative than Romer yet still reliable in our simulation.

Doing so, we found 9 significant miRNAs (hsa-miR-516b, hsa-miR-639, hsa-miR-503, hsa-miR-191*, hsa-miR-548a-5p, hsa-miR-300, hsa-miR-369-5p, hsa-miR-431*, and hsa-miR-200a*). Interestingly, these do not overlap with the miRNAs that were reported to be differential by Gupta et al. However, looking at the GO-terms (Table 6) associated with the target genes of these 9 miRNAs similar categories as originally reported appear. These are mainly cell cycle and transcription activity, such as ‘S Phase’, ‘nucleosome’, ‘nucleoplasm’ or ‘nucleosome assembly’ in the top ranks, but also ‘telomere maintenance’ which is rather apoptosis-related. Besides, terms related to blood cell functions (‘negative regulation of megakaryocyte differentiation’ and ‘blood coagulation’) appear.

## Discussion

Studies on the molecular aspects of a wide range of diseases now focus on relations between mRNA and miRNA expression. Besides the two above-analysed examples, parallel expression levels of both types of molecular features were also studied in studies on colorectal cancer [33], medulloblastoma [34] or colon polyps [35]. In a large simulation study we show that combining high-throughput miRNA and mRNA expression data improves the power of testing either data type individually. In most simulation settings, combined testing yielded higher power rates than classical miRNA-wise testing or target set testing alone.

Apart from that, the threat of false positives in gene set testing is lowered by our approach. In particular, the liberal behavior of the global tests is somewhat diminished by the combination approach. However, their self-contained character leads still to many false positives, especially in the case of overlapping gene sets. In this context, it should be remarked that our scores proposed in the methods section do not allways behave completely like 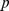-values. In our simulation studies the score based on Stouffer’s method was approximately uniformely distributed in the interval [0, 1] under the complete null hypothesis, i.e. when no group effects were introduced. However, the score was scewed to the left when being based on Fisher’s method. That means, regarding the score as a 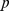-value is almost appropriate with Stouffer’s method but leads to somewhat too concervative results with Fisher’s method. Nevertheless, the proposed scores are a useful tool to rank the studied miRNAs according to their relation to the experimental grouping factor. Of course, the application of those procedures that did not maintain the pre-specified 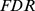-levels (as shown in Tables 1 and 2) is not recommendable for feature selection. Based on these findings, our future plans involve the development of some transformation rules for the scores, so that 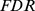-procedures also work for those procedures for which the pre-specified 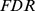 level was not yet maintained.

In most cases, the competitive approaches deal better with the problem of overlapping gene sets. The enrichment approaches remained close to the 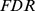-level desired to be controlled in our analyses. Naturally, they are more robust to gene set overlaps. In our simulations the enrichment-based approaches were also robust to the inter-gene correlations, i.e. they behaved similar under the different correlation structures we simulated. Nevertheless, one should keep in mind that under certain correlation structures their 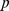-values may become skewed even under the null hypothesis. A simple solution would be to apply a sample permutation procedure, given that, unlike in our simulations and data example, there are enough replicates to show low 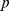-values.

The rotation-tests control the FDR in a non-overlapping gene set context. Otherwise, they profit from the combination in that they control the FDR better than the respective gene set tests – ‘Romer’-based combined testing even controlled the 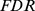 in all our simulations. To achieve this they lose power, however.

By applying our procedure on real microarray data we show its usability in everyday research. Especially the rotation test-based procedures are able to differentiate between miRNAs which were differentially expressed with little result in their gene set and others that lead to differentially expressed targets. For miR-290, for example, they successfully included the information from the miRNA’s gene set. There, they were able not to detect a miRNA which has little effect on its target gene set.

Furthermore, many new miRNAs were detected. Even for the most conservative procedure 21 further miRNAs were found to show an effect between E11 and E13. Since we have shown in our simulations that our combination approach maintains a pre-specified FDR in most cases, we belief that most of our positive findings in the data example are true positives. Therefore, we regard it as an improvement that we find more significant miRNAs by our combination approaches than were found in the original analysis by miRNA-wise testing alone.

We outlined information combination in two-group testing. To generalise our approach to three or more groups is not a hard thing to do. Both ‘limma’ for miRNA-wise testing, as well as the gene set tests presented can be used for any number of groups or continuous response variables. Indeed, arbitrary design matrices have already been implemented in the ‘miRtest’ package.

So far the procedure suggested needs, strictly speaking independent 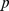-values from miRNA- and mRNA-data. The Fisher- and inverse normal method have originally been designed for independent repetitions of experiments. An example for matched data would be that the same individuals were taken for miRNA and mRNA microarray analysis. Such designs are not too infrequent. An idea to cope with that would be to jointly permute or rotate the expression matrices of miRNAs and mRNAs.

Another point is to include strategies to overcome gene set overlaps, i. e. the strong positive correlation between the miRNA-test statistics (to correct for multiple testing according to [36] appears to be too rigorous and ignores the information one has on overlaps). Ideas on how to control the FDR with this problem exist for gene set testing. See for example approaches for the GO graph in [37] or [38]. It appears worthwile to include such ideas for miRNA-testing in future work. Finally, apart from p-value combinations, one can also consider other ideas from meta analysis in the context of combining results from different microarray experiments. One could for example combine effect measures like the fold change by means of the inverse normal method. However, it seems to be not reasonable to employ meta-analytic methods for combining effect measures, in the context of our approach, since there is up to now no established measure describing the up- or down-regulation in the global test setting.

In summary, we developed a method to seek out miRNAs that show an effect either in their own expression, or in their respective gene set between two groups. Our method enables researchers to analyse miRNA data in a more statistical reliable manner than to test miRNA-expression and mRNA-expression separately. As miRNAs directly act on their mRNA targets miRNA-mRNA interactions compose a quite simple bipartite network. Its incorporation into testing for differential expression via gene set tests helps to gain power. On the other hand, miRNA expression data leads to less type I errors.

The algorithm was implemented in the ‘miRtest’ R package available via CRAN (http://cran.r-project.org). As competitive approaches performed better in our analyses, we chose the ‘Romer’ gene set test as a default and recommend the Wilcoxon test for those who want to apply a less time-consuming algorithm.
